# A review of e-textiles in neurological rehabilitation: How close are we?

**DOI:** 10.1186/s12984-016-0167-0

**Published:** 2016-06-21

**Authors:** Ruth McLaren, Frances Joseph, Craig Baguley, Denise Taylor

**Affiliations:** Health and Rehabilitation Research Institute, Faculty of Health and Environmental Science, AUT University, Private Bag 92006, Auckland, 1142 New Zealand; CoLab: Creative Technologies Research Centre, Faculty of Design and Creative Technologies, AUT University, Private Bag 92006, Auckland, 1142 New Zealand; Faculty of Electrical and Electronic Engineering, AUT University, Private Bag 92006, Auckland, 1142 New Zealand

**Keywords:** E-textiles, Electronic textiles, Smart fabrics, Rehabilitation, Telerehabilitation, Conductive elastomers, Knitted piezoresistive transducers, Functional electrical stimulation, Transcutaneous electrical stimulation

## Abstract

Textiles able to perform electronic functions are known as e-textiles, and are poised to revolutionise the manner in which rehabilitation and assistive technology is provided. With numerous reports in mainstream media of the possibilities and promise of e-textiles it is timely to review research work in this area related to neurological rehabilitation.

This paper provides a review based on a systematic search conducted using EBSCO- Health, Scopus, AMED, PEDro and ProQuest databases, complemented by articles sourced from reference lists. Articles were included if the e-textile technology described had the potential for use in neurological rehabilitation and had been trialled on human participants. A total of 108 records were identified and screened, with 20 meeting the broad review inclusion criteria. Nineteen user trials of healthy people and one pilot study with stroke participants have been reported.

The review identifies two areas of research focus; motion sensing, and the measurement of, or stimulation of, muscle activity. In terms of motion sensing, E-textiles appear able to reliably measure gross movement and whether an individual has achieved a predetermined movement pattern. However, the technology still remains somewhat cumbersome and lacking in resolution at present. The measurement of muscle activity and the provision of functional electrical stimulation via e-textiles is in the initial stages of development but shows potential for e-textile expansion into assistive technologies.

The review identified a lack of high quality clinical evidence and, in some cases, a lack of practicality for clinical application. These issues may be overcome by engagement of clinicians in e-textile research and using their expertise to develop products that augment and enhance neurological rehabilitation practice.

## Background

The increasing miniaturisation of electronic circuitry is facilitating the development of new and diverse technologies for rehabilitation. These include on-body sensing systems to measure limb movement, pressure and temperature, and the associated communication circuitry to enable data transfer. As these products have increased in ubiquity they have been proposed for rehabilitation, for example, with researchers applying accelerometers to measure functional movement of stroke patients, and smart functional electrical stimulation systems to elicit muscle contraction during function [1–5]. As the technology has progressed conventional electronic components have been integrated into clothes, including bend sensors placed in pockets over joints to measure movement [6], pneumatically driven cells embedded into orthoses [7–9] and accelerometers attached to clothing [10]. A further extension of this work is the development of electronic textiles, also known as e-textiles, whereby the electronic circuitry is embedded within the textile, rather than attached to the textile.

For the purpose of this review an e-textile is defined as a textile with electronic properties, or components integrated into the fabric, enabling the textile to perform sensing and, or, actuating functions [11, 12]. The potential advantages of e-textiles are significant, and a decade ago scientists speculated that e-textiles would change the face of rehabilitation [13]. This belief arose from the vision that e-textiles could be integrated into garments to allow sensing and actuating functions to be carried out over long periods of time in an unobtrusive manner. Proposed uses included activity monitoring, personal coaching and feedback, ambient sensing [14], gait analysis, analysis of dyskinesia [15], electromyography (EMG) sensors to control active orthotics, prostheses and mobility assistive devices [16, 17], outcome measurement [15], assessment of falls risk [10], monitoring functional ability at home, and providing electrical stimulation [18]. In light of the number of articles proposing such uses for e-textiles we aimed to determine if there was any substantial evidence to support the use of e-textiles in the rehabilitation of people with neurological conditions. For this review we placed no limits on outcome measures, comparison group, or study design.

To realise the advantages of e-textiles for neurological rehabilitation patient and therapist acceptance is critical. This involves design factors such as a garment being lightweight, soft, flexible, washable and wearable. At the same time, e-textiles must be able to perform and function accurately and effectively in a clinical sense. Accordingly, the review critically evaluates the usability and potential effectiveness of e-textile garments when used for neurological rehabilitation applications. Based on the review shortcomings are identified and future research directions recommended.

### Search method

**Fig. 1:**
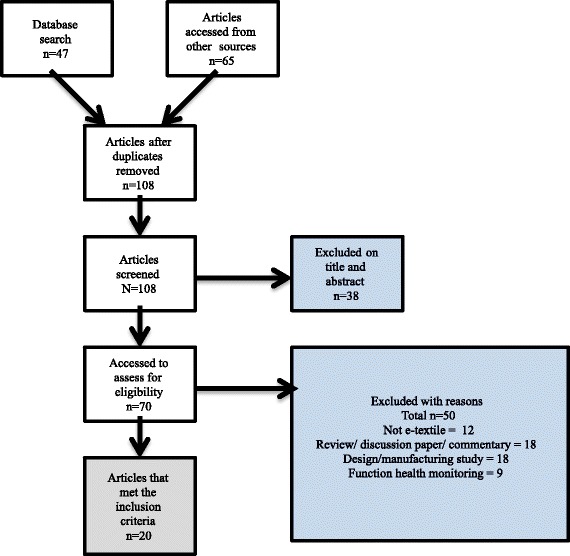
Flow chart of the paper selection process and results

A systematic electronic document search using the PRISMA guidelines [19] where possible was conducted in October 2015 of the EBSCO- health, Scopus, AMED, PEDro and ProQuest databases. Using key words “intelligent textile*” OR “intelligent fabric*” OR “e textile” OR “e fabric*“OR “e-textile*” OR “e-fabric*” or “electronic textile*” OR “electronic fabric*”. We did not exclude any design, outcome measure or comparison group. Further articles were accessed from reference lists and reviews on the topic. After duplicates were removed there were 108 articles. Articles were then screened by their title and abstract for relevance looking for reference to rehabilitation, physiotherapy, functional electrical stimulation (FES), motion analysis, prosthetics, falls, healthcare, biomechanics, biomedicine, or telemedicine. After screening by title and abstract we excluded 38 articles. Full text for the remaining 70 articles was accessed. Articles were included if they used some element of e-textile technology (including e-textile components sewn onto fabric or an item of clothing), if the e-textile had potential to be used for assessment or rehabilitation for people with a neurological deficit and if the e-textile had been tested on a human subject. Due to the limited numbers of papers and the multidisciplinary nature of the research no research study design was specifically excluded. However, articles were excluded if they were reviews, discussion or commentary on e-textiles, if they did not use e-textile technology (most commonly these were studies using functional technology or wearable electronics), if the article focussed solely on the process of engineering, fabric, or clothing design and/or manufacture, or if the e-textile’s primary function was health monitoring (Fig. 1.). Some articles fitted into more than one category for exclusion. If there was some ambiguity whether the articles met the criteria or not they were assessed by a second author and any disagreements were resolved by discussion. Twenty articles met the inclusion criteria and have been included in this review. It is worth noting that as e-textiles are an emerging field of technology and design available commercially, researchers with commercial links may have subsequent restrictions on publications of their findings, therefore, there may be some risk of bias in the studies found. Commercial products that are currently available include the Sensoria smart running sock [20], the StretchSense textile sensor [21], and the Teximat pressure mat by Texisense [22]. However, there are also collaborative projects such as the MyHeart and Wealthy projects developing e-textiles with high commercial potential that may have publication limits placed on them by funders [23].

Data was collected regarding, study design, aim, type of e-textile, the number and type of participants, the intervention, outcome measures used and the main findings. As many of the research papers that met the inclusion criteria were prototype design and validation studies, only research questions and data that met the inclusion criteria were included in the review and in the summary of results. Aspects of the papers that involved engineering and design of components and laboratory testing procedures have not been reported on as this is outside the scope of our research question.

## Results

A total of 108 records were identified and screened with 20 papers meeting the inclusion criteria (Fig. 1). There were no randomised controlled trials, controlled studies or case reports providing substantial clinical evidence for the efficacy of e-textiles in neurological rehabilitation. Of the 20 papers included 14 papers primarily focussed on prototype design and validation [16, 24–36] and four focussed on sensitivity and specificity testing of an already existing design [37–40]. There were two pilot studies in healthy populations [41, 42] and a clinical pilot study was reported as part of one paper [38].

Movement recognition has been the primary focus of e-textile research in neurological rehabilitation with 18 papers having been published in this area [24–33, 35–42]. Two papers have been published investigating e-textiles and muscle function [16, 34]. Nine studies used conductive elastomer e-textile technology [25, 26, 30, 32, 36–41]. Six studies used a knitted sensor [24, 27, 29, 31, 33, 35] one study used a stainless steel yarn electrode [16], one elastic conductive webbing [28], and one a screen printed multilayer electrode system [34]. One paper described their sensors as E-textile sensors but did not give further information on the design of the sensors [42]. Despite the papers looking into the use of e-textiles and their relevance to neurological rehabilitation only in one small pilot was the e-textile ever trialled on an individual with a neurological impairment [38]. Table 1. provides a summary of the findings of these 20 papers.

**Table 1:** Summary of research on e-textile development for neurological rehabilitation

Study	Study design	Aim	Type of e-textile	Participants	Intervention/device	Outcome measures	Main findings
Tormene, 2012, [32]	Prototype design and validation	Trunk motion data from e-textile garment.	CE	1 healthy subject	Trunk movts	CE and intertial sensor readings.	Same accuracy as inertial sensor in sagittal plane.
Mattmann, et al., 2007, [41]	Feasibility/Pilot study	E- textile shirt to classify body postures	CE	8 Healthy males	1. Sensing shirt worn during 27 postures 2. Worn during trunk rotation exercise	1. E- textile sensor data and observation. 2. E- textile sensor data.	1. 25/27 postures classified with 97 % accuracy after 6 reps. 80 % accuracy when 3 reps and 65 % for a new user. 2. Can distinguish 4 grades of speed and reps.
Lorussi et al. 2004, [36]	Prototype design	E- textile sensor to monitor arm position	CE	Not reported	Subject wearing sensing sleeve pointing at targets	Comparison between calculated position of arm and true position	Relative error between true and calculated position 4-8 %
Tognetti, 2005, [30]	Prototype design and validation.	Sensing shirt to measure UL movement.	CE	Not reported	1. Measuring UL posture. 2. Measuring UL movement.	1. Avatar posture, expert opinion. 2. CE, electrogoniometer readings.	1. 100 % accuracy. 2. Divergence at some angles. Some loss of synchronisation.
Giorgino, Tormene, Lorussi, et al. 2009, [37]	Intersubject and inter- exercise variability.	Wearing an e-textile shirt. 1. Intersubject variability. 2. Interexercise variability.	CE	1. 3 healthy subjects 2. 1 healthy subject	1. Shoulder flexion. 2. Three UL exercises.	1. CE sensor readings 2. CE sensor readings.	1. There was low intersubject variability. 2. Each exercise showed clear variability in the pattern of results.
Giorgino, Tormene, Maggione, et al., 2009, [38]	1. Sensitivity and specificity testing 2. Pilot rehab study	1. Sensitivity and specificity of a sensorised shirt. 2. Acceptability of sensorised shirt.	CE	1. 1 healthy subject 2. 13 sub acute stroke patients	1. UL exercises performed. 2. Rehab device used on ward.	1. CE sensor readings, expert opinion. 2. 10 Qualitative questions.	1. Three shirts had adequate sensitivity & specificity. Refined sensor position had better results. 2. Good acceptability for users
Giorgino, Tormene, Maggioni, Pistarini, et al., 2009, [39]	Sensitivity and specificity testing	Evaluate sensitivity and specificity of a sensorised shirt.	CE	1 healthy subject	7 UL exercises.	CE sensor readings, expert opinion.	Exercises that stretch a fabric can be reliably classified.
Giorgino et al., 2007, [25]	Prototype design	1. Develop e- textile system that classifies exercises for neuro rehab. 2. Between session variability of the sensorised shirt.	CE	1. 1 healthy subject 2. 2 healthy subjects	1. 11 UL rehab exercises. 2. 11 UL rehab tasks. Shirt doffed; donned after 1 h. Exercises repeated.	CE sensor readings.	1. Redesign resulted in greater differences between readings. 2. 7 of 11 exercises were classified incorrectly when shirt was reapplied.
Lorussi et al., 2005, [26]	Prototype design and validation	1. Develop sensing glove that recognizes hand positions. 2. Recognize novel hand posture.	CE	20 healthy adults	1. Calibrated glove 32 hand postures repeated randomly. 2. Novel posture of hand held.	1. CE sensor 2. CE sensor, not stated.	1. 100 % recognition. 98 % recognition if removed and worn again. 2. Average error measuring joint angle 4 %.
Cabonaro et al 2014, [24]	Prototype design and validation	Compare e-textile motion sensor glove with optical tracking.	KPF sensors	5 healthy subjects	Repeated natural hand movts.	KPF sensor readings, optical tracking system.	Accuracy of glove slightly less than commercial electrogoniometer.
Preece et al., 2011, [27]	Prototype design and validation	1. Investigate output of KPF sensor in a sock, during walking. 2. Feasibility of predicting gait events using sock with KPF sensor.	KPF sensor	20 healthy adults	Walking wearing instrumented sock; shod and unshod.	KPF strain sensor, 3D video gait analysis.	1. Graphed sensor values and kinematic signals show similar characteristics. 2. Accurate HL & TO predicted offline HS prediction less accurate.
Sung et al. 2009, [29]	Prototype design and validation	Identify human movement during walking and running using e-textile sensors.	Knitted stainless- steel yarn sensor	5 healthy male adults.	Walking and running wearing e- textile suit.	e-textile sensor readings.	Similar results running & walking. Increased speed; individual habits insignificant.
Yang et al., 2010, [33]	Prototype design and validation.	Develop e-textile sensor system to monitor movts and posture.	20 Knitted sensors	Not specified.	Fast walking, slow walking & falling down.	E-textile sensor readings.	Sensor signal patterns differed for each condition.
Shu, et al., 2010, [35]	Prototype design and validation	Design e- textile sensor to monitor plantar pressure during gait	Knitted conductive sensor coated in silicon	8 healthy males	Subject wearing sensing innersole stepping and standing	Sensor CoP during standing, one leg stand, heel strike and push off compared to CoP on force plate.	CoP relative difference Standing 7.9 %, One leg stand 9.9 %, Heel strike 0.5 %, Push off 2.2 %.
Tognetti et al. 2014, [31]	Prototype design and validation.	Compare KPF goniometers with electrogoniometers and inertial measurement units.	KPF sensors.	Not specified	KPF sensor over knee joint. One legged sit to stand at varied speeds.	KPF sensor, inertial measurement unit, electrogoniometer.	The KPF goniometer followed dynamic knee movts (maximum error 5°).
Shyr et al., 2014, [28]	Prototype design and validation	Measure the flexion angle of elbow and knee movts.	Elastic conductive webbing	1 healthy adult	Repetitive elbow and knee flexion/extension.	Protractor, e-textile sensor	Good relationship between e-textile sensor and joint angle.
Munro, et al., 2008, [40]	Reliability and validity	E- textile sensor to control audible biofeedback of movement pattern.	CE	5 female and 7 male athletes	Intelligent knee sleeve worn during hopping and stepping activities	Kinematic data, and audible feedback signal compared knee angle (goniometer)	Able to reliably distinguish between shallow and deep knee flexion.
Helmer et al., 2011, [42]	Pilot study	E- textile sensor to 1. measure knee movement and 2. Trigger auditory biofeedback to change kick pattern	Not specified	Not specified	E- textile sensorised leggings worn during kicking.	E- textile sensor data compared to 3D video analysis	1. Reliably measured max knee flexion during kicking < 10 % error 2. E- textile triggered audio signal. Change in kicking pattern post biofeedback training.
Farina et al., 2010, [16]	Prototype design and validation	Design electrode grids for recording EMG.	Stainless steel yarn electrodes	3 healthy subjects	Static postures of the hand and wrist.	EMG readings from e-textile.	Tasks classified with accuracy of 89.1 % +/- 1.9 %
Yang et al., 2014, [34]	Prototype design and validation	Design screen- printed fabric electrode array to stimulate muscle.	Multi- layer screen printed electrodes.	2 healthy individuals	E-textile/PCB array stimulated to produce hand postures.	Electrogoniometer	E-textile >90 % of movt generated by PCB array. E-textile greater repeatability.

*Abbreviations*: *CE* conductive elastomer, *COP* centre of pressure, *HL* heel lift, *HS* heel strike, *KPF* Knitted Piezoresistive Fabric, *max* maximum, *movt* movement, *movts* movements, *neuro rehab* neurological rehabilitaiton, *PCB* printed circuit board, *rehab* rehabilitation, *ROM* Range of motion, *TO* Toe off, *UL* upper limb

### Movement recognition

Following a neurological injury a common impairment is loss of the ability to move with sufficient force and control of the body or body segment. One of the aims of rehabilitation is to enable a person to perform day-to-day activities in a timely and controlled manner with appropriate muscle activity. Movement recognition technology has the ability to provide information to patients and therapists on the degree, quality and dose of movement. This data may be used to trigger an actuator, provide feedback during daily life or during a set exercise session, or enhance the experience of rehabilitation. A common means of sensing movement is through the use of piezoresistive materials. In e-textiles these materials are primarily made of conductive fibres that are woven, knitted or embroidered into fabric, or are constructed using silicon embedded with a conductive material and screen printed onto fabric [14, 43, 44]. When the sensors are mechanically deformed, the electrical resistance increases and then decreases to a steady state value, returning to baseline when the strain is removed [30]. The basic supposition of e-textile sensors is that when the user holds a certain posture, the sensors produce a resistance value related to that posture. Thus, if sufficient sensors are appropriately placed, a particular set of resistance values will characterise a unique posture [24, 45, 46].

#### Trunk posture

Mattmann et al. [41] used a conductive elastomer sensor thread embedded into a commercially available close fitting suit. They used this sensorised shirt to classify 27 body postures. When the posture was repeated 6 times the garment was able to distinguish 25 of the 27 postures with 97 % accuracy. When the posture was repeated only three times accuracy dropped to 80 %. The postures that were less reliably distinguished were all positions that had similar torso positions but were performed sitting or standing. This suggests that the positon of the sensors in this garment were not suitable to distinguish between sitting and standing. To explore the feasibility of this garment in a real world situation this garment was donned by a subject who then performed dynamic resisted torso rotations. The garment was able to distinguish amplitude of movement, speed of movement and number of repetitions.

Tormene et al. [32] investigated conductive elastomers printed onto a corset to measure specific trunk movements. The garment was able to reliably distinguish trunk flexion as small (30°), medium (60°) and large (90°) when movements of these different amplitudes were performed at varying speeds. They also performed trunk extension and left and right side bending, however; the sensor configuration was not sensitive enough to reliably distinguish those movements. The limitations of these studies [32, 41] highlight the importance of considering the functional movements that are to be measured and positioning sufficient numbers of sensors in a manner to ensure their maximum distortion, and thus optimal performance for the target movements [46].

The garments created by Tormene et al. [32] and Mattmann et al. [41] are sensitive enough to provide general postural information suitable for monitoring trunk movement during an exercise programme, feedback on the number of repetitions and perhaps some gross information on the level of movement control as measured by the speed of motion. However, for other rehabilitation tasks involving smaller trunk movements such as the degree of trunk rotation, side flexion or pelvic tilt during gait or balance related tasks, increased sensitivity is required. Further work needs to be done to determine the clinical utility of devices such as these.

#### Upper limb posture

Lorussi et al. [36], proposed distributed sensing systems based on the direct deposition of strain sensors onto fabric. The strain sensors were piezo-resistive types, realised through coating elastic fabrics with conducting polymer and also with carbon filled rubber. Prototype gloves incorporating strain sensors were developed, as well as sleeves to fit over the length of an arm. A method of reading measurements from a distributed sensing system was given, and experimental work verified the proposed system could locate shoulder and elbow angular posture positions to within an 8 % error. This early paper presented the possibilities of e- textiles in a positive light warranting further investigation.

Tognetti et al. [30] created an upper limb-sensing garment made from Lycra printed with conductive elastomer sensors. Prototype validation initially seemed very positive. A healthy volunteer wearing the garment performed 50 different arm positions which a computer generated avatar was able to replicate with 100 % accuracy [30]. The subject removed the garment, then put it on again and undertook another series of testing. This subsequent testing on 16 postures resulted in 90 % accuracy without any further calibration.

The researchers then performed testing to assess whether the avatar could copy novel postures that weren’t included in the calibration set of 50 postures, by comparing the avatar position with data from electrogoniometers. This showed significant divergence at some parts of the range of motion and a lack of synchronisation between the electrogoniometers and avatar [30]. It was hypothesised that the non-linear response to stretch, was responsible for the lack of accuracy when measuring novel movement patterns. An additional problem was, the higher the velocity of movement the greater the overshoot in resistance change and the longer it took to return to the steady state value. This could be a wait of up to two minutes holding the target posture [30].

To overcome the issues measuring real time movement the upper limb sensing garment was further developed for clinical use by improving the sensor layout, weighting the importance of different sensors and adjusting the algorithms used [25, 38]. The goal was to accommodate the time dependent characteristics of the sensors and recognise arm position during movement. Performing a simple glenohumeral shoulder flexion exercise the information gain from the sensor signal of one subject correlated well with sensor signals from three healthy adults [37]. The data from three different exercises performed by one person showed unique patterns of sensor readings for each exercise [37]. This round of development of the garment improved the specificity of movement recognition (correct vs. incorrect movement pattern) with the best of the three prototypes having a sensitivity of 89 % +/-6 % and specificity of 93 % +/-5 % [37].

However, though the prototype garment was able to reliably measure a static posture without recalibration, to reliably sense a dynamic movement pattern the garment required calibration before each session [25, 30]. Data analysis was also more accurate, sensitive and specific when assessed off line in a time series that was clipped to only include the event being studied, compared to real time analysis. Open-ended real time movement patterns are currently unable to reach the accuracy predicted by computer generated models [39]. These factors limit the use of this garment in a fluid real life situation. However, the technology appears suitable to monitor movement during specific exercises or to monitor general movement patterns. This information may be used give feedback, apply principles of gamification to improve patients engagement and motivation in rehabilitation, monitor progress or provide information to therapists remotely.

The reliability of the garment to sense a desired movement pattern has also only been tested in a healthy subject who mimicked the type of incorrect movement patterns a person may make following a stroke [25, 37–39]. Further research is required using this system on individuals with a central nervous system lesion to assess the responsiveness of the e-textile sensor system to movement patterns affected by impairments such as spasticity, and reduced dexterity inherent in this population.

A feasibility study has been conducted assessing the acceptability of a shirt embedded with conductive elastomer sensors to provide remote rehabilitation for 13 patients following stroke [38, 39]. The shirt was calibrated before each session giving the advantage of making it very easy for therapists to prescribe, customise and progress exercises as the patient improved and giving the flexibility to design a programme that is meaningful to the patient. However, a disadvantage of this regular calibration is the time involved each session and the dependence on the patient and carers understanding of the desired movement pattern. A patient satisfaction survey found that the sensorised shirt was generally acceptable, easy to understand and considered useful by patients [38]. Whilst the data was not specifically reported, the researchers mentioned that the patients who best related to the system expressed a perceived improvement from its use. However, patients with lower cognition (mini mental state examination between 23 and 27) had problems handling the system and perceiving improvement [39]. In general, the reported results indicate that patients found the e-textile sensorised shirt was an acceptable and feasible adjunct to rehabilitation [38, 39]. Disappointingly, no outcome measures looking at motor ability or functional status were made. Therefore, we cannot draw any conclusions regarding the sensing garments usefulness as a rehabilitation tool.

The concept of being able to use an upper limb-sensing garment to provide remote rehabilitation or to augment a current exercise programme is appealing. In the upper limb, research suggests that a higher intensity of rehabilitation exercises may produce a more favourable result, and a relatively high dose of repetitive functional tasks to develop and improve motor planning and motor function has also been advocated [47]. An upper limb sensing garment has the potential to promote these aspects of rehabilitation in a way that gives the patient autonomy whilst remaining under the supervision of a therapist [39]. However, for this potential to become a reality, further research is required to gauge the sensing garment’s effectiveness as a therapeutic tool, and also to consider its reliability and validity when assessing movement patterns in an individual with a central nervous system lesion.

#### Hand posture

Hand gestures have been assessed using both conductive elastomer and knitted piezoresistive sensors. Conductive elastomer sensors printed onto a glove have had similar results to the conductive elastomer sensing upper limb garment previously described [30]. There was 100 % recognition of defined static finger postures that had been calibrated for the subject. Posture recognition dropped only slightly to 98 % after the glove was doffed and donned [26]. However, as for the upper limb garment, non linear transient time related properties of the sensors required holding particular postures for up to 25 seconds to gain a steady state at which reliable detection could be made [26]. While the ability of the glove to recognise a static posture is excellent, the time needed to hold the posture and gain this information makes it impractical for use in a clinical scenario to measure functional activity. However, a sensing glove with these capabilities may be of use as a form of feedback or short term monitoring during a set exercise programme.

Tognetti et al. [31] developed a double layer knitted piezoresistive sensor that measures changes in resistance to bending but not to elongation. This type of sensing layer was then incorporated into a glove [24, 31]. The piezoresistive sensors were calibrated to an optical tracking system and had a measurement accuracy of +/- 3.6° [24]. The main discrepancy in measurement accuracy occurred when the speed of movement altered quickly, such as the acceleration and deceleration when a movement changed from flexion to extension. This could be due to a similar dependence of transient time length on velocity, to that reported for the conductive elastomers [26]. These promising results were tempered by some practical limitations of the technology. Currently, using these sensors to measure one degree of freedom at one joint requires 8 connecting pads and 6 wires. This limits their use in joints with multiple degrees of freedom or small joints. The authors propose that configuring the sensors for multiple joints in series will improve the feasibility of this system in practice [24, 31]. In the future it would be good to see how sensors with this degree of sensitivity respond to analysis of functional movement at multiple joints, for example using the sensorised glove to analyse different functional grasps and also the reliability and sensitivity of movement analysis in people who have altered movement patterns from a neurological impairment.

The use of an e- textile glove has been proposed as a means of facilitating and providing feedback and motivation to complete more therapeutic exercises. It has also been suggested that an e-textile glove would be useful to gauge the true measurement of hand use over a day and promote more day-to-day functional activity, something that therapists struggle to achieve currently [24]. However, wearing a glove may impair the sensory experience of movement and touch. Hand sensation is critical to the control of hand function, therefore, wearing a glove can impair the dexterity of the hand during both gross movements and fine motor tasks. In addition, a glove worn in functional day to day situations will get dirty and would have to be removed to be washed which may limit the motivation to use a gloved limb in many basic functional tasks such as eating, personal hygiene activities, preparing food, and cleaning [48]. Currently, these issues have not been well addressed, and are areas in need of further research work to determine the acceptability, reliability and usefulness of a glove as an assessment and rehabilitation tool.

Many day to day tasks involving the arms are bimanual. Therefore it is also conceivable that a person with a hemiplegia could use a sensing glove of this nature on their affected limb to control an actuator or robot. This could provide the function of one hand whilst leaving the unaffected limb free to complete the other part of the task, increasing ease and fluidity of using assistive technology.

#### Gait analysis

E-textiles for gait analysis have been explored in four studies and three different directions have been taken for gait analysis. Firstly, through focussing on individual joint motion and looking to identify specific parts of the gait cycle as measured by the biomechanics at that one joint [27]. Secondly, through focussing on gait as a whole body movement and using information from multiple body sites to give a representation of gait [29, 33]. And thirdly by looking at the use of an innersole embedded with e- textile sensors to look at changes in the centre of pressure during gait [35].

Preece et al. [27] investigated a resistive strain sensor knitted into a sock to determine the degree of correlation that could be achieved between the sensor output and the ankle joint angle during a gait cycle. The sensor was implemented through knitting an electrically conductive yarn in a stitching pattern that caused the resistive path length, and thus resistance, to increase with increasing levels of strain [27]. Outputs from the knitted sensors within the sock were compared to kinematic data from 3D video gait analysis. When graphed, the sensor output from the knitted sensors showed similar features to the scaled kinematic data. However, the degree of match varied considerably between individuals and between the shod and unshod conditions. Despite this, it was possible to accurately identify when heel lift and toe off occurred with a mean error of 1- 1.6 % of the gait cycle and heel strike within 2.6 and 3 % of the gait cycle. This is an accuracy similar to using accelerometers to predict gait events and is acceptable in clinical practice [49]. However, the current gait model Preece et al. [27] have developed using the e-textile sock has several limitations. Firstly, it requires manual adjustment of sensor thresholds for each individual for the algorithm to work. Secondly, data needs to be analysed offline, rather than in real time therefore it can’t be used to predict gait events.

Whole body gait analysis using e-textiles was performed by Yang et al. [33] and Sung et al. [29]. Yang et al. [33], used 20 knitted sensors embedded within a shirt, a pair of trousers and socks to measure gait. Pressure data was gathered from the sensors based on pressure as the arm moved past the axilla, the elbow or knee bent past a certain angle, or the sensors on the foot we compressed by weight bearing and the sensors were determined to be either ‘on’ or ‘off’. Sensor signals showed different patterns for walking slow, fast and falling tasks [33]. A biomechanical model of gait was used to determine cadence, step length, gait speed and acceleration. Sung et al. [29] inserted eight braided e-textile piezoresistive sensors into a commercial sports suit. Interestingly, they choose points over muscles commonly used in gait and running, not necessarily the point of greatest stretch in the garment, which has shown better differentiation between movements in other studies [39, 46]. Their five subjects showed similar shaped curves in time/electrical resistance change during walking and running. There was less change in resistance for the trunk than for the lower body sensors as the trunk moves less than the legs during gait. As the speed of motion increased and subjects began running the slope of the curves increased and the stable periods reduced. This was due to increased acceleration and deceleration of body parts and increase in the difference between the maximum and minimum resistances. This also had the effect of reducing the impact of individual walking habits on resistance change [29].

Disappointingly, Yang et al. [33] and Sung et al. [29] primarily focussed on prototype design and did not assess the reliability of their calculations against another method of gait analysis. Therefore, although these systems of gait analysis look easy to use and don’t require a lot of computing, the reliability, validity and sensitivity of the systems and the usefulness in clinical practice remains unknown.

Shu et al. [35], investigated the use of 6 textile sensors embedded in an innersole to compare the centre of pressure during standing, stepping and walking. They were able to measure changes in pressure during these activities and also predict centre of pressure with reasonable accuracy at heel strike and toe off compared to a force plate. This soft and sensitive pressure sensor with its wireless data transmission to a variety of platforms for analysis shows potential for use not only in a research laboratory or clinic but also outdoors and for activity monitoring.

The concept of using an e-textile sensor to measure real time gait is an appealing one. The level of data that could be obtained in real life situations is considerable. From a therapeutic perspective, a common deficit following stroke is altered ankle kinematics and kinetics. Using an e-textile sensor to identify the heel strike or toe off phases of gait could enable us to control an actuator to functionally stimulate the ankle dorsiflexors subsequent to these phases of the gait cycle. This is significant in that these muscles have been identified as having great potential for functional electrical stimulation as an assistive technology or functional orthoses. Currently there are wearable electronic products available commercially that provide this function [50–52], however, the unobtrusive nature of e-textiles may provide a lighter more discrete and user friendly option for consumers. Smart fabrics could also be suitable for additional functions such as sensing that an individual was fatiguing and should rest before further activity, providing feedback on foot clearance, cadence, weight transfer, gait speed and distance walked.

#### Further lower limb analysis

Further research has been performed in a number of lower limb studies. Tognetti et al. [31] and Shyr et al. [28] have both investigated the development of new prototypes involving knitted and woven e-textile stretch sensors that have been validated during active movement at the knee joint. Tognetti et al. [31] have produced a double layer knitted sensor that is able to be calibrated quickly and has a maximum error of 5° during physiological movement at both fast and slow speeds [31]. Shyr et al. [28] do not provide information on measurement error during active movement for their woven sensor however graphical representation suggests that resistance change at the sensor correlates well with knee angle during knee flexion and extension.

Helmer et al. [42], and Munro et al. [40], have used e- textile stretch sensors to provide auditory biofeedback regarding the knee range of motion. Munro et al. [40] used an intelligent knee sleeve with conductive elastomer sensors during hopping and stepping tasks designed to emulate a sports person landing. While the actual knee angle the audible bio- feedback was triggered at was significantly different than the angle it was set to be triggered at, the intelligent knee sleeve was able to reliably distinguish between shallow and deep knee flexion. The authors conclude that for the functional task of landing this level of variability is acceptable but for tasks that require greater discrimination in movement this sensor may not be suitable. Helmer et al. [42], mounted an e- textile strain sensor across the anterior knee of sports training leggings and assessed it on a healthy subject. Compared to 3D video analysis they found the measurement error was less than their acceptable limit of 10 % and mapping of the data showed similar graphical representation. During the kicking task it was desirable for the subject to flex the knee greater than 85° flexion. The researchers used the e- textile to trigger an auditory signal when the knee reached a threshold of 85° flexion and used this biofeedback as training in kick technique. Immediately following this biofeedback training the graphical representation of the sensor feedback indicates that learning occurred and that increased knee flexion prior to ball contact occurred. The movement in these sports studies are performed at speeds unobtainable by our patients with neurological injury. However, they show potential for future applications using e- textile biofeedback to alter movement patterns and enhance learning.

### Muscle activity

To date research has focussed primarily on the development of e-textiles with sensing capabilities. However, some of the most intriguing possibilities of e-textiles are their potential to assist or perform biophysical functions. Functional electrical stimulation of muscles is one of the ways e-textiles may be used in the future, either as a functional orthosis or as a therapy tool. Farina et al. [16] investigated measurement of surface EMG via an e-textile. To this aim, they have developed a sleeve with stainless steel yarn electrodes sewn in a matrix pattern over the flexor and extensor muscles of the wrist and elbow. EMG readings for 9 functional hand and forearm movements were obtained. EMG analysis was able to discriminate between these functional movements with around 90 % accuracy [16]. Future research could look at incorporating this technology into a garment and investigating the potential of using EMG measured by an e-textile to trigger the delivery of functional electrical stimulation to a muscle.

Yang et al. [34] have developed a screen-printed flexible, breathable fabric electrode array. This dry electrode array was in direct contact with the skin and did not require a conductive hydrogel interface to stimulate a muscle contraction. This potentially made the electrodes more comfortable, durable and easy to apply. By stimulating the electrodes using pre-programmed patterns they were able to accurately and comfortably recreate three different functional hand gestures with less than a 7 % error across all joints for each of the three postures. This was greater accuracy than muscle stimulation using the leading alternative, a flexible printed circuit board with hydrogel layer. The significance of the difference between the two electrode types was not reported on, however, we can conclude that the e- textile electrode performed at least as well as the leading alternative in this situation.

Until recently, measuring and augmenting muscle function has required conductive pads, gels and wires. The current technology allowing the measurement and stimulation of muscle without use of conductive gel and via embroidered or printed electrodes that have become part of the fabric is the next step towards incorporating these elements seamlessly into a garment. Future research is required to explore how electrode placement and skin contact is maintained when the electrodes become part of a full garment, assessing the quality of the EMG data that can be obtained and using an e-textile sensor system to trigger a functional actuator response. This will increase the wearability and usability of EMG and muscle stimulation, giving them the potential to be used on a day-to-day basis in both therapy and real life situations, instead of the current situation where they are predominantly used for short periods of therapeutic intervention.

## Conclusion

For all the successful laboratory prototypes and potential that e-textiles offer there have been a number of technical and manufacturing obstacles to overcome. There remains a significant gap between the proposed roles e-textiles have in the future and what can be delivered at present. Notwithstanding these limitations, continuing developments in e-textile technology show that they have considerable potential yet to be realised.

This review has identified 20 articles that have applied e-textile interventions in a way that could promote neurological rehabilitation. Currently there is no substantial evidence to support the use of e- textiles in neurological rehabilitation. Due to the heterogeneous study designs, variation in prototypes that are being assessed, outcome measures used and small sample sizes synthesis of the results was not possible. However, some general conclusions can be drawn. To date, research has focussed primarily on motion sensing function in the therapeutic setting. E-textiles have shown promise in their ability to reliably measure gross movement and whether an individual has achieved a predetermined movement pattern. However, this has primarily been demonstrated for large, slow movements. The non-linear phenomena of hysteresis, transient time length and the effects of movement velocity on sensor readings have limited the use and accuracy of e- textile sensors in reconstructing and measuring fast and small movements [31, 45]. Advances in sensor design and the computing behind the scenes, will give us greater options for how we use these sensors and how data is assimilated [24, 27, 28, 33, 45]. New ways of using information on movement patterns that allow for the current limitations of fabric based sensors give us scope for further investigation into their validity in real life situations, biofeedback, and movement analysis and what this means to us as clinicians and as individuals [29, 32, 33]. Current research into the usability of e-textiles to measure EMG and provide FES through fabric based electrodes is in its infancy, however these concepts have opened up potential for where this technology may take us in the future [16, 34].

Whilst there are still a number of technical barriers to overcome in the development of e-textiles, initial prototypes look encouraging. One of the limitations of the e-textile literature in neurological rehabilitation is that engineers and biophysicists have led the majority of research to date with little input from clinicians. This has resulted in an emphasis on the evolution of engineering and technical processes needed to develop wearable sensors and systems [15]. As technological limitations are overcome it is becoming evident that to make the next step in technology development we need innovators from bordering professions such as rehabilitation therapists and designers to be exposed to some of these disparate areas outside of their experience, to expand their thought processes, and become involved in directing these innovative ideas into useful and usable adjuncts for rehabilitation [53].

Collaboration between engineers, biophysicists, designers and therapists in the development of e-textiles is well overdue. This partnership is required to assess the efficacy, reliability and validity of this technology in a population with a central nervous system lesion and to ensure that the e-textiles as a rehabilitation adjunct produce a clinically meaningful change. Furthermore, one of the most promising aspects of e-textiles is their ability to unobtrusively be worn in daily life. Therefore, there is potential for rehabilitation therapists, designers, engineers and also consumers to be involved in further development and research into actuating functions and everyday assistive technology.

## Abbreviations

EMG, electromyography; FES, functional electrical stimulation
